# EEG Biomarkers for a Precision-Medicine Approach to Noninvasive Brain Stimulation for Major Depressive Disorder

**DOI:** 10.21203/rs.3.rs-7771697/v1

**Published:** 2025-10-06

**Authors:** Rubén Romero-Marín, Davide Cappon, Javier Solana-Sánchez, David Bartrés-Faz, Álvaro Pascual-Leone, Gabriele Cattaneo

**Affiliations:** Institut Guttmann, Institut Universitari de Neurorehabilitació adscrit a la UAB, Badalona, Spain; Department of Neurology, Harvard Medical School, Boston, MA, USA; Institut Guttmann, Institut Universitari de Neurorehabilitació adscrit a la UAB, Badalona, Spain; Departament de Medicina, Facultat de Medicina i Ciències de la Salut, i Institut de Neurociències, Universitat de Barcelona, Barcelona, Spain; Department of Neurology, Harvard Medical School, Boston, MA, USA; Institut Guttmann, Institut Universitari de Neurorehabilitació adscrit a la UAB, Badalona, Spain

**Keywords:** eeg, tms, biomarkers, depression, nibs

## Abstract

Major Depressive Disorder (MDD) is a prevalent and debilitating psychiatric condition with significant rates of treatment resistance. Non-invasive brain stimulation (NIBS), including transcranial magnetic stimulation (TMS) and transcranial electrical stimulation (tES), has emerged as a promising option for individuals unresponsive to pharmacological interventions. However, a substantial proportion of patients still fail to achieve meaningful clinical improvement, underscoring the need for reliable biomarkers to predict treatment response. Electroencephalography (EEG) and TMS-EEG have been increasingly explored as promising predictive tools due to their ability to assess cortical excitability, connectivity, and neuroplasticity. The evidence gathered from 18 high-quality studies highlights the relevance of EEG and TMS-EEG biomarkers in predicting outcomes of NIBS in MDD. Resting-state EEG studies emphasize the importance of spectral power alterations, alpha asymmetry, and connectivity patterns, while TMS-EEG studies underline the role of TMS-evoked potentials (TEPs), particularly the N100 and N45 components, in forecasting therapeutic response. While these findings suggest significant potential, methodological variability, small sample sizes, and differing stimulation protocols limit their immediate clinical translation. However, these biomarkers provide a solid foundation for implementing precision medicine. Prior EEG or TMS-EEG assessments can play a valuable role in guiding the personalization of NIBS treatment strategies. The systematic integration of these neurophysiological biomarkers into clinical practice could maximize therapeutic efficacy and reduce non-response rates, paving the way for more precise and effective interventions in depression treatment.

## Introduction

Major depressive disorder (MDD) is a prevalent and disabling psychiatric condition, affecting approximately 280 million people worldwide (1). Despite the availability of various pharmacological and psychotherapeutic treatments, approximately 20–40% of patients suffer from chronic depressive episodes (2), fail to achieve meaningful relief and become medication-resistant (3–5).

Emerging evidence suggests that MDD symptoms arise from rapidly evolving pathological brain states within specific neural circuits (6, 7). Non-invasive brain stimulation (NIBS) techniques, including transcranial magnetic stimulation (TMS) and transcranial electrical stimulation (tES), can precisely target these circuits, disrupting the maladaptive, self-sustaining activity and – perhaps due to such mechanisms - alleviating depressive symptoms even in chronic cases (8–10).

Early clinical studies suggested that repetitive TMS (rTMS) targeting the dorsolateral prefrontal cortex is effective in medication-resistant cases (11), leading to rigorous clinical trials and eventually the clinical adoption and regulatory approval of rTMS for medication resistant MDD (12–14).

Current treatment protocols show that approximately 60% of patients experience significant improvement—with 30% achieving full remission after a six-week rTMS course (13, 15, 16), and accelerated protocols reaching remission in as little as one week (17, 18). However, approximately 40% of patients do not respond as expected to treatment. Poorer outcomes are especially common among those with more severe baseline symptoms (19), as well as in cases involving altered network connectivity and imbalances in neural excitability and inhibition (to be discussed in the following sections). These findings underscore the importance of adopting a precision-medicine approach (20) that adjusts treatment based on each individual’s biological profile to enhance therapeutic effectiveness and a greater understanding of NIBS mechanisms.

Electroencephalography (EEG), whether used alone or in combination with TMS (TMS/EEG), has emerged as a powerful tool for understanding the complex dynamics involved in the pathophysiology of MDD (21). This approach enables researchers to examine how alterations in neural circuitry contribute to depressive symptoms, offering valuable insights into the underlying mechanisms of the disorder. TMS/EEG leverages the focal stimulation capabilities of TMS alongside the high temporal resolution of EEG, allowing researchers to capture the immediate neural responses evoked by TMS (22, 23). When a single TMS pulse (spTMS) is applied, it depolarizes cortical neurons, triggering synaptic activations that are recorded as TMS-evoked potentials (TEPs) in the EEG. These responses provide direct, real-time measures of cortical excitability and connectivity, offering invaluable insights into the dynamic interactions within brain networks (24–30).

By characterizing the unique spatiotemporal and physiological profiles of individual patients, TMS/EEG can help identify predictive biomarkers that indicate who is most likely to benefit from NIBS. Such biomarkers could not only pave the way for personalized treatment strategies but also have the potential to reduce nonresponse rates, optimize therapeutic protocols, and lower the economic and clinical burdens associated with ineffective interventions (31).

Some have argued that EEG and TMS-EEG hold considerable promise as predictive biomarkers for MDD interventions (32–34).

In the present paper, we provide an updated review of the current literature on EEG and TMS/EEG biomarkers as predictors of NIBS response in MDD. While EEG-based measures and TMS-EEG metrics hold considerable promise as predictive biomarkers, further research is needed to validate their clinical utility. Through a systematic review of the literature, we identify both encouraging findings and major inconsistencies that underscore the need for more rigorous and standardized research. Our goal is to move beyond speculation and outline the specific gaps that must be addressed before EEG and TMS-EEG can reliably inform personalized NIBS interventions. Ultimately, we propose a framework of key neurophysiological determinants that should guide future investigations and help bring the field closer to clinically actionable biomarkers.

## Methods

### 3.1 Search strategy

PubMed and Scopus were searched between May 1st, 2024 and October 1st, 2024 for publications studying markers of treatment response in patients with MDD. Search terms for both databases were (Markers) AND (Treatment Response) AND (Depressive Disorder) AND ((EEG) OR (electroencephalography) OR (TMS) OR (NIBS) OR (tDCS) OR (tACS) OR (tES)). Results were filtered to only include studies reported in English with a date range between 2019–2024. Two study authors (RR, GC) conducted an independent literature search using pre-defined inclusion and exclusion criteria. Conflicts between authors were resolved by discussion. Approved studies were then moved to data extraction.

### 3.2 Inclusion criteria

The included studies focused on subjects with unipolar MDD who received noninvasive brain stimulation treatments. To be eligible, studies were required to report EEG or TMS-EEG measurements taken before, during, or after the treatment. Additionally, only studies that demonstrated a moderate or higher level of quality of evidence, as assessed following the selection process, were considered.

### 3.3 Exclusion criteria

Case studies, review articles, protocols, posters, and conference abstracts were excluded from the analysis. Studies involving animal populations, healthy subjects, bipolar depression, or conditions other than unipolar MDD were also excluded. Additionally, studies that did not include any therapeutic interventions or utilized pharmacological treatments instead of NIBS were not considered. Finally, only studies that reported EEG or TMS-EEG measures were included; those without these measurements were excluded.

### 3.4 Quality of evidence assessment

Quality of evidence was assessed using the **Grading of Recommendations Assessment, Development, and Evaluation (GRADE)** methodology (35). The quality of studies was categorized into four levels: high, moderate, low, and very low. Studies rated as ‘high’ were randomized, double-blinded, and placebo-controlled; ‘moderate’ studies were randomized but not blinded; ‘low’ studies were non-randomized but included a placebo or control group; and ‘very low’ studies were non-randomized without a placebo or control group. To ensure focus on higher quality and more reliable designs, studies rated as ‘very low’ were excluded (36).

## Results

### 4.1 Study selection

**Figure 1.**
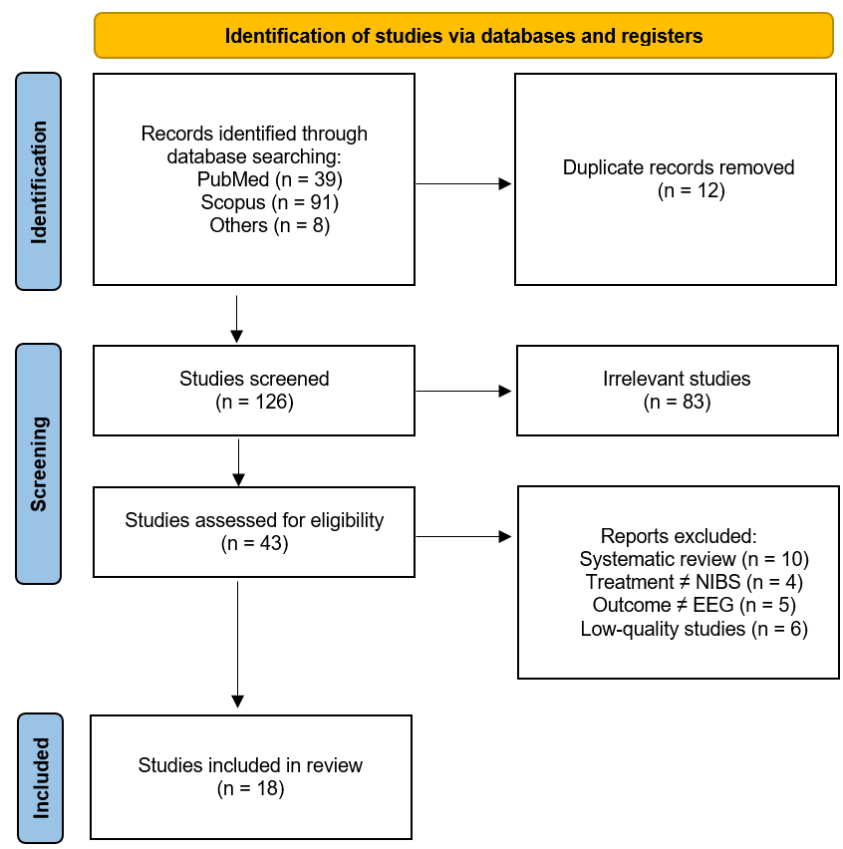
Provides detailed information on the study selection process, following the Preferred Reporting Items for Systematic Reviews and Meta-Analyses (PRISMA) guidelines (128).

A summary of the study selection process, including inclusion and exclusion criteria, is provided in Figure 1.

### 4.2 Included study characteristics

The search terms initially yielded 138 studies, which were reduced to 126 after removing duplicates. During the primary screening, 83 studies were excluded based on title or abstract for not aligning with the focus of the review, leaving 43 studies for full-text evaluation. Of these, 25 studies were excluded due to not meeting the inclusion criteria — for example, studies using neuroimaging modalities other than EEG or TMS-EEG, involving interventions not related to NIBS, or examining populations or outcomes outside the scope of this review. In addition, six studies were excluded following a quality assessment that rated them as “very low” due to methodological limitations such as small sample sizes, lack of control groups, or absence of randomization. Ultimately, 18 studies were included in the qualitative synthesis. Quality ratings for these studies are detailed in Tables 1 and 2. The following sections are organized chronologically by year of publication.

### 4.3 Resting EEG studies

Eleven studies explored EEG markers of treatment response to brain stimulation therapies (Table 1), utilizing a wide range of quantitative EEG measures.

Studies that explored the association between EEG power and the response to **repetitive transcranial magnetic stimulation (rTMS)** revealed that higher baseline power in different frequency bands was associated with increased treatment success. Specifically, different studies found that individuals with better responses to the rTMS intervention had increased beta power (37,38), higher alpha power (39,40), increased theta relative power (41), and low gamma power (40). Additionally an individual alpha frequency (IAF) closer to 10 Hz showed greater clinical improvement after 10 Hz rTMS (42). However, two studies showed contradictory results, showing an association between lower beta power (39), alpha power (43) and better clinical outcomes.

Among the reviewed studies, only two have investigated prefrontal theta cordance, both reporting significant findings differentiating responders from non-responders (see supplementary material for details) (37,38). And only one study examined EEG microstates, finding that rTMS treatment led to an increase in microstate MS-2 occurrence and coverage, and a decrease in MS-3 (44). However, this study did not assess whether microstate features could predict clinical response to rTMS.

In terms of connectivity, better response to the intervention was associated with increased alpha and beta band coherence (45), as well as decreased theta band coherence (46), and an increase of Alpha Spectral Correlation (αSC) after treatment (47).

Non-Linear Features combined with machine learning achieved an impressive accuracy predicting treatment response (37,38).

Increases in alpha current source density current source density (CSD) were also significantly associated with clinical improvement (45).

These examples illustrate the diversity of EEG metrics explored to date, with researchers often selecting specific features of interest rather than employing comprehensive or standardized approaches. While data-driven methods, including machine learning, offer promising avenues to identify complex biomarker patterns, they remain difficult to interpret and compare across studies due to high variability in input features, sample characteristics, and the algorithms applied. This heterogeneity poses a significant barrier to drawing clear conclusions about EEG-based predictors and calls for more standardized frameworks and validation efforts.

### 4.4 TMS-EEG studies

Seven studies have reported TMS-EEG markers of treatment response to NIBS therapies in MDD (Table 2), primarily using **TEP** components, which involve examining features such as peak amplitude, peak latency, and slope. Additionally, one study analyzed activity and connectivity patterns using **Significant Current Density (SCD)** and **Significant Current Scattering (SCS)** methods.

Six studies have examined the effects of therapeutic TMS on TEP components. Higher baseline N45 (48) and N100 (49,50) amplitudes were linked to improvements in depression symptoms. In contrast, a smaller baseline P60 component predicted better response to treatment (48).

Regarding the changes after treatment, a decrease in P30 (51), N45 and N100 (50,52) amplitude correlated with improved clinical outcomes. However, N45 results are inconsistent, as some have found an increase with treatment (50).

In terms of slope, treatment responders exhibited a steeper negative N100 slope for single pulses and a steeper positive slope for paired pulses at baseline compared to non-responders (53). Additionally, a decrease in SCS was found to be positively correlated with improvements in depression severity following rTMS (24).

## Discussion

This systematic review that synthesized the growing body of evidence on resting-state EEG and TMS-EEG biomarkers in depression—especially in treatment-resistant depression (TRD)—demonstrates both exciting promise and important challenges. Recent studies have begun to identify specific, quantifiable metrics that may predict treatment response to NIBS.

### 5.1 Key Findings and Overall Trends

#### Resting-state EEG

##### Alpha Band Power

Resting-state EEG studies consistently reveal alterations in spectral power and connectivity in depressed populations. For example, depressed individuals often exhibit heightened synchrony in the theta and alpha bands (54,55), a phenomenon that may reflect abnormal thalamocortical connectivity (56).

Multiple studies consistently report that greater baseline alpha power —especially over frontal and parietal regions— has been associated with better responses to rTMS (57–61). Some work suggests that increased alpha power may reflect lower arousal and reduced serotonergic activity (38), though discrepancies remain, with other studies failing to find significant differences or even reporting decreased alpha power in depressed cohorts (62–65). Further complicating the picture, post-treatment changes in alpha power are inconsistent, with some studies reporting decreases (55,66) and others noting increases (67). These mixed results likely reflect interindividual differences and methodological variations, such as pre-treatment stress levels influencing sympathetic activation.

##### Alpha Asymmetry and Entrainment

Evidence also points to the relevance of frontal alpha asymmetry, with left frontal regions often showing differential activity compared to the right. Since EEG alpha power is an inverse index of cortical activity (68), reduced alpha at right compared to left frontal sites (i.e., greater right-sided activation) has been associated with withdrawal motivation (69), negative affect (70–72), as well as reports of more intense negative emotions (73,74). In MDD, studies have shown that alpha activity in the left frontal region typically are lower than that of the right (75–77). This asymmetry has been linked to the dysfunction of the left DLPFC, a key target in depression treatment (78,79), and may help predict treatment outcomes depending on the stimulation site. Specifically, for rTMS targeted over the lateral prefrontal cortex (LPFC), greater left-sided alpha activity correlated negatively with clinical improvement, while stimulation over the medial prefrontal cortex (MPFC) showed a positive correlation with clinical improvement (40). This is consistent with PET studies reporting decreased frontal metabolism (80–83) and Voxel-Based Morphometry (VBM) studies revealing reduced gray matter volume in these regions (84), although the relationship between structure and cortical excitability remains to be fully clarified (85).

In addition, the observation that an IAF near 10 Hz appears to be optimal, suggesting that aligning stimulation frequency with the brain’s intrinsic rhythm (via entrainment) may enhance neuroplasticity and clinical outcomes (42,86). The Arnold tongue model provides a theoretical framework for understanding how the frequency and amplitude of external stimuli interact with intrinsic oscillations to promote synchronization (86). According to this model, entrainment peaks when external stimulation—be it rTMS or tACS—precisely aligns with the brain’s natural rhythm (87). This perfect synchronization drives powerful neuroplasticity, paving the way for lasting clinical and behavioral gains (42). However, inconsistent findings, likely due to individual differences in intrinsic alpha frequency (IAF) (47,88,89), and the sparse documentation of rTMS effects on these oscillatory patterns in MDD beyond isolated case studies (90,91), call for further investigation.

##### Other resting-state EEG frequency bands

Investigations into other frequency bands have also provided valuable insights. Elevated baseline beta power has generally been associated with poorer treatment outcomes, as it may indicate greater depression severity (37,59), although some findings suggest that increased beta could reflect preserved reward processing (92). Significant coherence observed in the beta band has been associated with cortical excitability, suggesting that increased baseline coherence in large-scale networks may predict better outcomes from rTMS treatment (45). Additionally, frontal delta power, for example, is lower in responders (37,39,59), suggesting reduced temporoparietal dysfunction that influences emotional arousal (93). High frontocentral theta activity at baseline is linked to poorer outcomes and greater depression severity (59,88,94), though some studies report no significant differences (37,38,95). Moreover, higher medial low-gamma power, on the other hand, predicts better outcomes—possibly reflecting enhanced attentional capacity and cognitive control (40,96–98). Furthermore, increases in baseline connectivity, particularly in the alpha and theta bands, were linked to better treatment outcomes (46), especially in fronto-parietal regions, in line with other studies (99). Theta activity in prefrontal areas has been associated with the default mode network, which plays a role in high-level control over perception (100,101). Lower theta connectivity was linked with better antidepressant outcomes, possibly reflecting reduced top-down control (102). Additionally, lower connectivity in the salience network, which helps in switching from the DMN to the CEN, correlated with antidepressant responses (46). Higher αSC among responders suggest increased functional coupling between brain regions (47). Lastly, theta cordance in the prefrontal cortex holds promise as a predictor of clinical improvement following rTMS treatment, although statistical significance has not been consistently observed across studies, likely due to variations in study designs, sample sizes, and rTMS protocols (37,38).

##### EEG Microstates and non-linear features

Clinical improvements correlated with changes in EEG microstates (MS) MS-2 and MS-3 after rTMS treatment, with MS-2 increasing and MS-3 decreasing in occurrence (44). These changes suggest that rTMS modulate large-scale network alterations (103). Specifically, MS-2 has been linked to BOLD activation in the dorsal ACC, inferior frontal cortices, and right insula (103), regions associated with the reward circuit and improvements in anhedonia (104). Conversely, MS-3 showed reduced occurrence post-TMS, involving frontal and parietal cortices implicated in attention and cognitive control networks (103,105). While depression is often linked to hypoconnectivity in cognitive control networks, some patients exhibit hyperconnectivity, potentially contributing to rumination, which correlates with the DMN (104,106).

Non-linear EEG features, when integrated with machine learning models, demonstrate strong predictive power, (37–39) yielding high predictive accuracies. These complex dynamics effectively capture subtle changes in brain activity associated with NIBS response in depression. Notably, the higher complexity dimension (CD) observed in non-responders (39) suggests increased dimensionality, reflecting a broader distribution of neural oscillations with lower synchrony (107). This elevated CD may indicate neural isolation (108) and disorganized spiking activity (109), signaling more atypical brain activity in non-responders compared to responders.

#### TMS-EEG Biomarkers and Cortical Inhibition

##### TMS-Evoked Potentials (TEPs)

The field of TMS-EEG biomarkers has yielded significant insights into TEP components, particularly emphasizing the N100 component. Higher baseline N100 amplitudes were predictive of greater improvements in depressive symptoms (49,50). The N100 component reflects GABA_B_ receptor-mediated slow inhibitory processes (49). Deficiencies in GABA (110–113) and reduced size and density of GABAergic interneurons in the DLPFC in MDD patients (114) suggest that patients with higher baseline N100 amplitudes may have more intact inhibitory circuitry, allowing greater responsiveness to stimulation. Conversely, a lower baseline N100 amplitude could indicate impaired inhibitory circuitry. However, a significant reduction in N100 amplitude post-treatment among responders, was also observed (49,50,52), and the implications of these findings are in contradiction with the theory of GABA deficiency. Steeper negative slope for single pulses and a steeper positive N100 slope for paired pulses in responders (53), potentially driven by larger P60 amplitudes in the left hemisphere, suggests an interplay between GABA_B_ receptor-mediated inhibition and glutamatergic receptor excitation, potentially reflecting sensory processing differences of the TMS pulse (53). The N100 slope has been associated with cognitive performance in working memory (115,116) and has also been linked to antidepressant response in bipolar depression, with changes indicating long-term potentiation effects related to sleep deprivation (117).

Larger baseline N45 amplitudes correlating with improved clinical outcomes (48), indicating that greater cortical inhibition might enhance therapeutic effects through synaptic modulation and improved balance between excitation and inhibition. Post-treatment increases in N45 amplitude were noted among responders (50), suggesting enhanced GABA_A_ receptor-mediated inhibition, and a more robust and functional network of GABAergic interneurons enhances synaptic modulation capacity, facilitating therapeutic effects by modulating cortical excitation and maintaining a balance between cortical excitation and inhibition. An increased baseline level of inhibition in the left DLPFC may create a neurophysiological environment that is more responsive to the synaptic effects of treatment, potentially facilitating changes in neuronal plasticity (48). These modifications may help normalize cortical activity in patients with depression. However, these findings contrast with results related to the N100 component, suggesting that different inhibitory mechanisms may contribute to symptom improvement. The N45 component is linked to GABA_A_ receptor activity (118,119) and may also be modulated by the glutamatergic system (118), whereas the N100 component is associated with GABA_B_ receptor activity (119). If changes in N45 amplitude reflect the dynamic interplay between cortical excitation and inhibition, particularly due to its sensitivity to NMDA receptor antagonists and positive allosteric modulators of GABA_A_ receptors (118,119), this could explain the contrasting responses of N45 and N100 in treatment responders. Evidence indicates that an imbalance between cortical excitation and inhibition plays a key role in MDD pathophysiology (6,120,121). Thus, alterations in N45 and N100 amplitudes observed in treatment responders may reflect rTMS-induced restoration of this balance (50). However, opposite findings (52) show a decrease in N45, suggesting concordance with the N100-associated changes seen in symptom improvement. This points to potential complexity in how these biomarkers respond to treatment, indicating multiple pathways to clinical improvement.

The P60 amplitude, indicative of excitatory glutamatergic neurotransmission (122) showed that lower baseline amplitudes predicted greater clinical improvement (48), aligning with the hypothesis that reduced excitatory neurotransmission could indicate a more favorable treatment response due to heightened cortical inhibition. This aligns with observations of increased N45 amplitudes and improved outcomes, suggesting that a robust inhibitory system may support symptom improvement.

A decrease in P30 amplitude following rTMS (51), suggest that treatment may reduce prefrontal intracortical inhibition, which could contribute to clinical improvements in depressive symptoms due that that 10 Hz prefrontal stimulation suppressed the intracranial P30 evoked response (123,124), particularly in the stimulated network. Reduction in P30 amplitude may indicate a decrease in GABA-Aergic inhibition (119). Thus, rTMS might promote neural facilitation by decreasing prefrontal cortical inhibition. However, P30 amplitude is also associated with glutamatergic excitatory neurotransmission (116,125). Consequently, the reduction in P30 amplitude could suggest that a decrease in excitability might facilitate cortical inhibition. When considered alongside the observation that a stronger inhibitory system is linked to symptom improvement in depression, this suggests that a less robust inhibitory system may also function more effectively under conditions of reduced excitability.

Reductions in the effective connectivity signal (**SCS**) correlated with symptom improvement in depression (24), point to the possibility that subgenual cinguale cortex (SGC) and DLPFC interconnectivity may be partly modulated by GABAergic neurotransmission (126). The DLPFC plays a central role in MDD and is the primary target in many depression treatments. Several studies have focused on the DLPFC, measuring baseline and post-treatment changes due to the association between depressive symptoms and disrupted activity and connectivity in brain regions involved in mood and cognition (7). Specifically, the left DLPFC has been consistently linked to depression symptomatology, as it is typically hypoactive in depression; an increase in its activity often corresponds with antidepressant response (127). Conversely, the right DLPFC tends to be hyperactive in MDD, contributing to dysregulated connectivity and control within the limbic system (7).

### 5.2 Gaps in Knowledge and Critical Evaluation

These findings indicate that both resting EEG and TMS-EEG provide direct measures of cortical excitability and inhibition, offering valuable mechanistic insights into how NIBS modulates brain circuits in MDD, and suggesting promising biomarkers for treatment response. Differences in alpha and beta power, and certain (inhibitory’) TEPs are especially intriguing, with N100 and N45 predicting responses to treatments.

Despite the encouraging findings. Many studies suffer from small sample sizes, diverse study designs, and varying NIBS protocols, complicating cross-study comparisons and limiting the generalizability of results.

Small sample sizes in some studies lead to inconsistencies in results, and the diversity in study designs, NIBS protocols, and outcome measures complicates cross-study comparisons and limits the ability to draw firm conclusions. Specifically, the influence of pharmacological treatments, because each patient with depression takes a different treatment, and each treatment has different mechanisms of action that can cause EEG activity alterations and interfere with the neuronal response to NIBS. This is a confounding factor that we must take into consideration when interpreting results of the different studies. Also, the absence of standardized methods for EEG and TMS-EEG analysis further restricts the generalizability of these findings. Although some biomarkers show promise in predicting treatment response, their clinical application remains uncertain due to a lack of validation in independent cohorts and real-world settings. Additionally, there is an imbalance in the distribution of NIBS techniques among the included studies. Despite conducting a comprehensive search encompassing all NIBS modalities, most of the identified studies utilized rTMS, while tES was comparatively underrepresented. This disparity limits the generalizability of our findings across different neuromodulation approaches. Future research should aim to determine whether the identified markers are replicable and valid across all NIBS techniques to enhance their broader applicability. Additionally, further studies might also explore less-tapped EEG measures (e.g. like dynamic connectivity patterns) to uncover new insights, and should aim to conduct larger, more standardized studies that integrate multimodal approaches, including EEG, TMS-EEG, and neuroimaging, to create a more comprehensive understanding of treatment response. Developing reliable, individualized predictive models will be essential to advancing personalized treatment strategies for depression. Moreover, longitudinal studies examining the effects of NIBS on EEG and TMS-EEG biomarkers could provide insights into how brain networks adapt over time and how these changes correlate with long-term clinical outcomes.

### 5.3 Framework of Key Determinants for Future Investigation

To move toward more targeted and effective NIBS interventions for MDD, future research should focus on the following key determinants:

- Conducting larger, multicenter studies with standardized protocols and analytical methods to validate promising biomarkers across diverse populations and real-world settings.
- Incorporating longitudinal designs to evaluate how NIBS-induced changes in EEG and TMS-EEG biomarkers correlate with long-term clinical outcomes.
- Exploring the interaction between NIBS-induced neurophysiological changes and individual genetic, epigenetic, and neurochemical profiles to identify personalized treatment predictors.
- Enhancing computational modeling and machine learning approaches to integrate multimodal data (e.g., EEG, fMRI, behavioral measures) and refine predictive models of treatment response.
- Investigating the role of brain network dynamics in NIBS efficacy, focusing on functional and structural connectivity alterations that mediate antidepressant effects.

In conclusion, resting-state EEG and TMS-EEG offer unique and clinically relevant insights into the neurophysiological mechanisms underlying MDD and treatment response. Rather than remaining exploratory tools, these modalities should be systematically incorporated into the clinical workflow to guide NIBS interventions. Addressing current methodological challenges and advancing multimodal integration will further strengthen their role in enabling precision medicine, ultimately improving treatment outcomes and reducing non-response in depression care.

## Supplementary Material

Supplementary Files

This is a list of supplementary files associated with this preprint. Click to download.

• 2SA1.docx

## Figures and Tables

**Figure 2.**
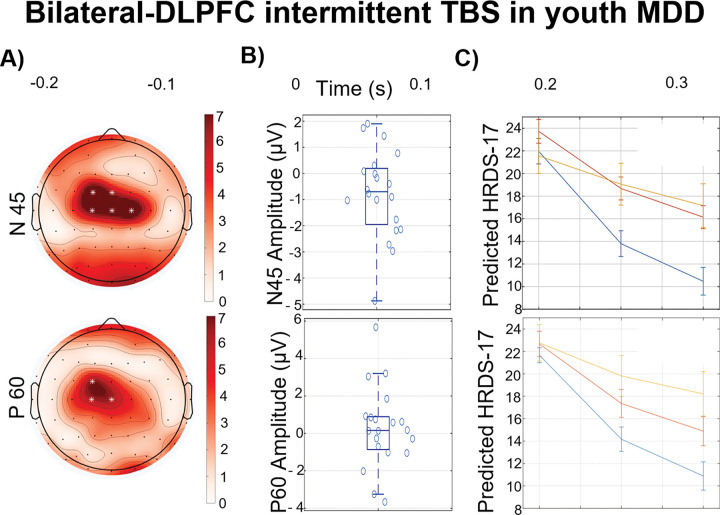
Baseline TMS-EEG markers predict response to bilateral DLPFC intermittent TBS treatment for youth depression (2 groups receiving respectively 2-week TBS, 10 sessions, or 4-week TBS, 20 sessions applied bilateral DLPFC. A) Topoplot analysis displays the p-values of the TEP × time interaction, highlighting significant electrodes with a white asterisk. B) Boxplot shows the distribution of amplitude values across participants for an exemplary electrode. C) Predictive line plots split participants into three groups based on their TEP amplitude, illustrating their predicted treatment response trajectory. Error bars indicate standard error of the mean. Adapted from Dhami et al., (2023) (48).

**Figure 3.**
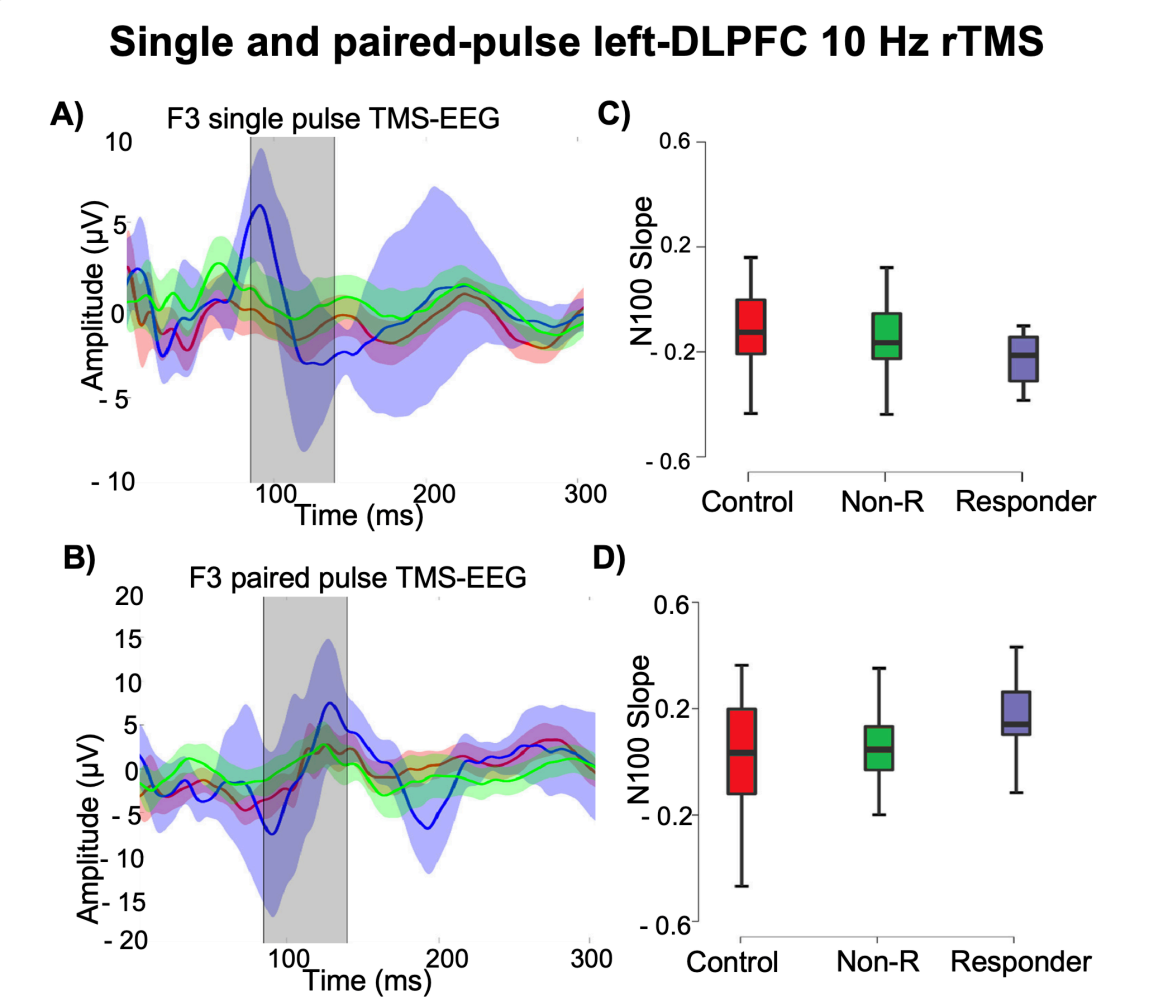
TMS-EEG markers (single and paired pulse) of response to 6 weeks of active 10Hz or sham rTMS targeting the left DLPFC or bilateral DLPFC. A) TEPs from F3 in response to single pulse LDLPFC stimulation from each group at baseline (red controls, blue responders, green non-responders). B) TEPs from F3 in response to paired pulse LDLPFC stimulation from each group at baseline (red controls, blue responders, green non-responders). C), D) Steeper N100 slopes in responders suggest that cortical inhibition dynamics may distinguish treatment responders from non-responders. Adapted from Bailey et al., (2023) (53).

**Table 1:** Resting EEG outcomes in brain stimulation studies include the type of treatment and EEG measures, baseline findings (capturing data before treatment initiation or during the initial stages), and post-treatment findings (reflecting changes observed after treatment completion). These included power analysis (such as absolute and relative power, IAF as well as FAA), cordance and microstates. Additionally, various connectivity metrics, including envelope correlation and αSC and coherence, were employed to assess inter-regional brain synchronization. As well as Non-linear features, such as PE, FD, LZC, CD, and KFD, were also included.

References	N	Treatment	Quality assessment	Measures	Baseline significative findings	Change in measure post treatment
Ebrahimzadeh et al., 2024	106	rTMS	Moderate	Power Analysis; Cordance; Non-Linear Features	↑β power; Combined features	-
Godfrey et al., 2024	28	rTMS	Moderate	Power Analysis; Connectivity	↓θ connectivity	θ connectivity↑
Ebrahimzadeh et al., 2023	88	rTMS	Moderate	Power Analysis; Cordance; Non-Linear Features	↑β power; ↓CD, ↓LZC	β power↓, CD↓, LZC↓, θ cordance↓
Zangen et al., 2023	169	dTMS	High	Power Analysis	↑α left; ↑Low-γ power; Combined features	-
Gold et al., 2022	49	rTMS	Moderate	Microstates	-	MS-2↑, MS-3↓
Voetterl et al., 2021	39	rTMS	Moderate	Power Analysis	↑θ relative power	α absolute power↑
Hasanzadeh et al., 2019	46	rTMS	Moderate	Power Analysis; Non-Linear Features	↓β power; ↑α power; ↑CD	-
Corlier et al., 2019	109	rTMS	High	Connectivity	-	αSC↑
Roelofs et al., 2019	153	rTMS	Moderate	Power Analysis (IAF)	IAF ≈ 10Hz	-
Alexander et al., 2019	32	tACS	High	Power Analysis	-	Left α power↓ (10 Hz tACS only)
Cook et al., 2019	16	sTMS	High	Power Analysis; Connectivity; Non-Linear Features	↑α coherence; ↑β coherence	CSD↑

**Table 2:** TMS-EEG outcomes for brain stimulation studies

References	N	Treatment	Quality assessment	Measure	Baseline significative findings	Change in measure post treatment
Sheen et al., 2024	23	rTMS	Moderate	TEP	↑N100	-
Dhami et al., 2023	43	TBS	Moderate	TEP	↑N45; ↓P60	
Strafella et al., 2023	98	iTBS	Moderate	TEP	↑N100	N100↓; N45↑
Bailey et al., 2023	39	rTMS	Moderate	TEP	↑sp ↓pp N100 Slope	-
Voineskos et al., 2021	30	rTMS	High	TEP	-	N45↓; N100↑
Eshel et al., 2020	33	rTMS	High	TEP	-	P30↓
Hadas et al., 2019	26	rTMS	High	Connectivity	-	SCS↓
